# Clinical and Prognostic Significance of Idiopathic Left Bundle-Branch Block in Young Adults

**DOI:** 10.1155/2021/6677806

**Published:** 2021-03-09

**Authors:** Pietro Delise, Luigi Rivetti, Giuseppe Poletti, Monica Centa, Giuseppe Allocca, Nadir Sitta, Arianna Cati, Giovanni Turiano, Emanuela Lanari, Paolo Zeppilli, Luigi Sciarra

**Affiliations:** ^1^Division of Cardiology, P. Pederzoli Hospital, Peschiera Del Garda, Italy; ^2^Division of Cardiology, Conegliano Hospital, Conegliano, TV, Italy; ^3^Studio Poletti, Bologna, Italy; ^4^Division of Cardiology, Hospital of S. Donà di Piave, S. Donà di Piave, Italy; ^5^Sacred Heart Hospital, Rome, Italy; ^6^Casilino Hospital, Rome, Italy

## Abstract

**Aims:**

LBBB is rare in healthy young adults, and its long-term prognosis is uncertain.

**Methods:**

56 subjects (aged <50 years), in whom an LBBB was discovered by chance in the absence of clinical and echocardiographic evidence of heart disease, were collected in a multicenter registry.

**Results:**

69% were males. Mean age at the time of discovery of LBBB was 37 ± 11 years. Mean QRS duration was 149 ± 17 m sec and 35% had left axis deviation. All patients had a normal echocardiogram, except for left ventricular dyssynchrony; 37 patients underwent coronary angiography (30) or myocardial scintigraphy during effort Eriksson and Wilhelmsen (2005), and in all cases obstructive coronary artery disease was excluded. In 2/30 patients who underwent coronary angiography, an anomalous origin of the CX artery from the right coronary sinus was found. Thirty patients underwent cardiac magnetic resonance; in 60% it was normal, while in 40% it revealed late enhancement, which in 33% was localized in the basal septum, suggesting fibrosis of the left bundle branch. During follow-up (12+/10 years, median 10 years) no sudden death occurred. At the end of follow-up, all patients were alive, except for one who suffered accidental death. Two patients (3.5%) underwent PM implantation owing to syncope. The echocardiogram at the end of follow-up revealed LV dysfunction in only one patient.

**Conclusions:**

In young adults without apparent heart disease, LBBB is a heterogeneous condition. In the vast majority of cases, the prognosis is good and no ventricular dysfunction occurs over time. However, as only 18% of our patients were aged >60 years at the end of follow-up, we cannot establish the prognosis in older age-groups.

## 1. Introduction

Left bundle-branch block (LBBB) is common in patients with heart disease, and in this clinical setting it may progressively lead to left ventricular dysfunction and worsen the prognosis [1–5]. By contrast, LBBB is very rare in young individuals without clinical evidence of heart disease. In these latter cases, its clinical and prognostic significance is unclear [6–10].

Some epidemiological studies have evaluated the prognostic significance of LBBB in subjects aged 50 years or more over follow-up periods of up to 28 years [6–8]. However, the results of these studies are controversial. Indeed, some authors [6] have suggested a negative prognostic value of LBBB with regard to the incidence of coronary artery disease, myocardial infarction, AV block and sudden death, while others [8] have suggested a good outcome, at least in patients with LBBB without left axis deviation.

In subjects aged less than 50 years, few studies are available. These have been conducted in unselected populations, and all have suggested a poor prognosis in comparison with subjects with right bundle branch block (RBBB) and controls [9, 10]. For example, in a study of USAF flying personnel (involving over 237,000 subjects) [9], 125 individuals with LBBB were discovered (119 aged less than 50 years) and 101 of these did not have overt diseases. Over a 10 year follow-up, 15% developed a heart disease or hypertension, and 9% died. These studies, however, were published many years ago and the absence of heart disease was established mainly on a clinical basis. It follows that the pathogenesis and prognostic significance of idiopathic LBBB in the young is poorly known.

In the present study, we analyzed a group of asymptomatic subjects, without clinical or echocardiographic evidence of heart disease and without other systemic or pulmonary diseases, in whom LBBB was discovered by chance before the age of 50 years. The aim of the study was to establish the prognosis of LBBB in young patients during a long-term prospective follow-up. In a subgroup of patients, we also investigated the possible causes of LBBB by means of coronary angiography and/or cardiac MRI.

## 2. Methods

Over several years, 56 consecutive subjects were collected in a multicenter registry by the cardiology institutions contributing to this study. The following inclusion criteria were applied:

- LBBB discovered incidentally before the age of 50 years, during routine ECG (performed during examination for sporting activity, before a surgical procedure, etc.);
- No clinical history of hypertension, pulmonary or systemic diseases such as sarcoidosis, or heart disease;
- No symptoms such as syncope, pre-syncope, dyspnea, chest pain;
- Normal echocardiogram (including atrial and ventricular volumes, ventricular thickness and ejection fraction), with the exception of septal and left ventricular dyssynchrony.

In all cases, the following anamnestic elements were investigated:

- Family history of heart disease;
- Family history of pacemaker (PM) implantation;
- Family history of Brugada Syndrome (BrS);
- Family history of LBBB, before or after 50 years of age;
- Family history of juvenile (<50 years) or non-juvenile sudden death (SD).

In all cases, a number of echocardiographic parameters were also calculated, in order to evaluate diastolic and systolic LV function and inter- and intraventricular dyssynchrony of the LV [11]. Early transmitral (E) and late transmitral (A) inflow velocities were obtained from the mitral inflow Doppler signals. Mitral annulus velocities were obtained from the septal annulus of the LV by means of tissue Doppler imaging (TDI). A pulsed-wave Doppler was used to evaluate the interventricular mechanical delay (IVMD). The right and left ventricular pre-ejection intervals were measured from the onset of the QRS on the ECG to the onset of pulmonary and aortic outflow; the IVMD was calculated by subtracting the pre-ejection intervals of the right ventricles from those of the left ventricles. Intraventricular dyssynchrony was evaluated by calculating the time delay between the motion of the septum and the left posterior wall contractions (SPMWD) on M-mode images from the parasternal short-axis view at the papillary muscle level. To evaluate the longitudinal LV dyssynchrony, apical 4-chamber views were used and the regions of interest (ROIs) were in the basal segments of the left ventricular lateral and septal walls. The LV dyssynchrony index (LVdys)—standard deviation of the time from cardiac cycle onset to minimum systolic volume - was assessed quantitatively as the maximum time delay between the two basal segments, and was calculated as the time interval from the onset of QRS to the peak systolic velocity (Sm).

Although no patient had symptoms suggesting a coronary artery disease, the direct study of coronary arteries (by coronary angiography or CT coronary angiography) was proposed in order to seek a possible cause of LBBB. If this was refused by patients, an imaging test of ischemia was suggested as an alternative (myocardial scintigraphy at rest and during effort). In all cases, cardiac MRI, before and after gadolinium, was also proposed. Not all patients agreed to undergo coronary angiography and/or cardiac MRI. 9 patients (7 asymptomatic, 2 with syncope or pre-syncope) underwent EPS to evaluate conduction intervals (AH, HV) and the induction of supraventricular and ventricular tachycardias, on the decision of single centers.

All patients were prospectively followed up by means of periodic clinical and echocardiographic examinations. Follow-up duration was calculated from the date of discovery of LBBB according to anamnesis. During follow-up, 48/56 patients (86%) were not on medical therapy. The remaining initiated ace-inhibitors for mild hypertension [5] or metformin for diabetes [3]. In all cases, a further echocardiogram was obtained at the end of follow-up. During follow-up, the following events were considered: syncope, death (including sudden death), heart failure, hospital admission for any reason.

### 2.1. Statistical Analysis

Continuous and categorical variables are expressed as mean ± SD and median (IQR), respectively. Echocardiographic data on enrollment and at the end of follow-up were compared by means of the 2-tailed unpaired *t*-test (for normally distributed data) or the Mann-Whitney *U* test. A *P*-value of <0.05 was considered statistically significant.

## 3. Results

The baseline characteristics of the patients are summarized in Table 1.

A total of 56 patients were enrolled, 38 of whom (68%) were males. Their mean age at the time of discovery of LBBB was 37 ± 11 (median 40 years). Thirty-four subjects (61%) were practicing various sports at a competitive (21) or non-competitive [12] level; in these cases, LBBB was discovered during pre-participation screening.

When first discovered, LBBB during serial ECGs was intermittent (tachycardia-dependent) in 16 cases (28%) and stable in the remaining 40 (72%). In 37 (66%), LBBB had an AQRS < −30°, while the remaining 19 (34%), had a left axis deviation (QRS axis ≥ −30°. Mean QRS duration was 149 ± 17 msec. In all cases, PR was normal.

Eight patients (14%) had a family history of ischemic heart disease. A relative of one patient with familial ischemic heart disease had died suddenly at the age of 55 years. One patient (1.7%) had a family history of dilated cardiomyopathy. Five patients (8.9%) had a family history of LBBB (3 before 50 years, 2 after 50 years), 2 of whom (3.5%) also had a family history of sudden death (SD) (at 51 and 61 years). One patient (1.7%) had a family history of PM implantation at the age of 72 years. In summary, 3 patients (5.3%) had a family history of SD: one of them also had a family history of ischemic heart disease, while the other two had familial LBBB.

Echocardiographic evaluation of the LV (Table 2) showed normal volumes (though at the upper limit of the range) and normal systolic function (though with EF at the lower limit of the range). LV diastolic function (evaluated in terms of E and A cm/s) was normal. The main anomaly, as expected, was left ventricular dyssynchrony during contraction (expressed by pre-ejection interval of LV, interventricular mechanical delay, septal to posterior wall motion delay) as a consequence of the conduction disturbance, with a delay of contraction of the LV in relation to the RV and septal dyssynchrony in relation to the postero-lateral wall.

Thirty-six patients (64%) agreed to undergo coronary angiography [13], CT coronary artery angiography [9] or myocardial scintigraphy at rest and during effort [6].

**Figure 1:**
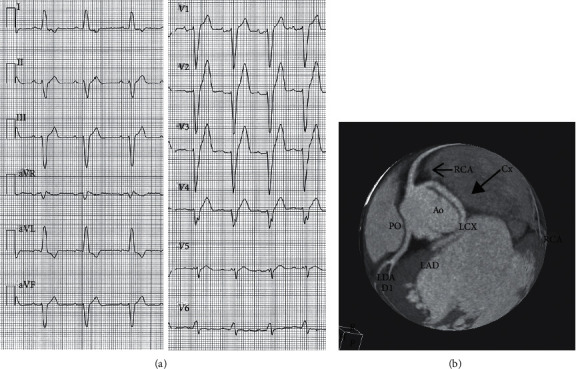
Male, soccer player. LBBB with left axis deviation discovered by chance at the age of 19 years. An ECG performed 2 years earlier was normal. The echocardiogram was normal. CT coronary angiography (performed at the age of 22 years) shows the left circumflex (CX) artery arising from the right coronary sinus, close to the right coronary artery (RCA). The CX runs back to the aorta.

In 27 (93%) of the 29 patients who underwent coronary angiography or CT coronary artery angiography, coronary arteries were normal. In 2/29 (7%) an anomalous origin of the circumflex artery from the right coronary sinus, running back to the aorta, was observed (Figure 1). In addition, in 4/39 (13.7%), a myocardial bridge on the left anterior descending artery was also detected, which was short and less than 2 mm deep. Of the patients who refused coronary angiography or CT, seven underwent myocardial scintigraphy (at rest and during effort), which was normal.

**Figure 2:**
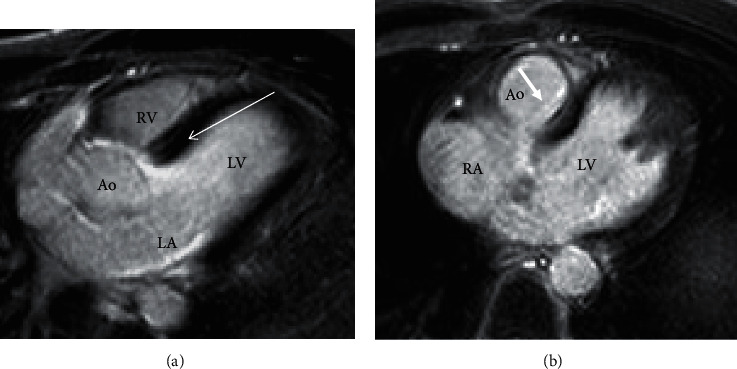
(a) and (b) Two males, aged 42 (a) and 48 (b) years, with idiopathic LBBB (a and b). In both cases, MRI shows late enhancement in the left posterior septum (arrows), possibly due to fibrosis involving the left bundle. Ao = aorta, RV and LV = right and left ventricles, respectively, RA and LA = right and left atria, respectively.

Twenty-nine patients (52%) agreed to undergo cardiac MRI, before and after gadolinium. In 18 cases (62%) MRI was normal, while in 11 (38%) it revealed late enhancement (LE) in the left ventricle. Specifically, in 10/11 (83%) LE was localized in the basal septum (Figures 2(a) and 2(b)). In one patient, LE was transmurally localized in the apex of the left ventricle. No patient with LE on MRI had a family history of LBBB or SD.

In 23 patients (41%) 24 hour Holter monitoring was carried out at least once. In no case were episodes of AV block recorded. In 3 cases (13%) > 100 supraventricular premature beats/24 hours were recorded, and in 4 patients (17%) > 100 ventricular premature beats/24 hours were recorded. In no case was non-sustained or sustained ventricular tachycardia recorded.

Electrophysiological study was performed in 9 patients (16%), which showed normal AH and HV intervals, and no supraventricular or ventricular tachycardias were induced by premature atrial and ventricular stimulation.

Follow-up data are summarized in Table 3. Follow-up duration from the date of the first detection of LBBB was 12 ± 10 years (median 8 years). At the end of follow-up, the median age of patients was 48 years, and 10/57 (18%) were 60 years old or more. No patient was lost to follow-up.

At the end of follow-up, 53/56 (95%) patients had stable LBBB on the basal ECG, including 13 who had had intermittent LBBB at the time of discovery of LBBB. During follow-up, 25/34 (74%) of the subjects who were practicing sport at the time of enrollment continued their sporting activity. Moreover, no SD occurred. One patient died as a result of an accident at the age of 47 years. One patient (1.7%) developed dilated cardiomyopathy with heart failure at the age of 69 years and underwent CRT-D implantation. Two patients (3.4%) reported syncope or pre-syncope at the age of 54 and 64 years, respectively. One of them underwent MRI which was normal. Both patients, with negative EPS, received a PM, which was programmed in the VVI mode at 40 min. During follow-up, neither of them became PM-dependent and both were paced for <1%. Another patient (1.7%) had a myocardial infarction at the age of 64 years. No patient with LE on MRI had adverse effects (syncope, PM implantation, SD or heart failure). In particular, no patient developed sarcoidosis.

At the end of follow-up, in 55/56 (98%) the echocardiogram was similar to the basal echocardiogram, showing normal left ventricular function and persistence of the signs of LV dyssynchrony (Table 2). In 1/56, who developed dilated cardiomyopathy (see above), a low ejection fraction (30%) was detected.

## 4. Discussion

LBBB is rare in young subjects (<50 years) in the absence of heart disease. In a large series of unselected young athletes (32,652 subjects), Pelliccia et al. [12] found a prevalence of 0.06%.

Little is known about the pathogenesis and prognostic significance of LBBB in young adults without heart disease. With regard to idiopathic LBBB in the young, a number of possible causes may be hypothesized: familial progressive cardiac conduction disease (Lenegre Disease) [14], concealed coronary artery disease [4], primary dilated cardiomyopathy, myocarditis [15] etc.

The prognosis of juvenile idiopathic LBBB is unclear, although some data suggest that it is negative. For example, in the USAF study [9] involving young subjects with LBBB without heart disease, 15% developed a heart disease or hypertension during follow-up and 9% died. Furthermore, LBBB might progress to AV block and/or cause sudden death [14]. In addition, on the basis of animal models and of clinical observations in humans [15–17], some authors have suggested that LBBB may progressively cause an induced cardiomyopathy by provoking an electromechanical dyssynchrony of the left ventricle.

In the present study, we analyzed 56 cases, collected in a multicenter registry by our cardiology institutions over many years, in which LBBB was discovered incidentally during routine ECG. None of these subjects had any clinical or echocadiographic evidence of heart disease. Many of them underwent a number of instrumental evaluations (coronary angiography, cardiac MRI etc.) in order to discover the possible causes of the conduction abnormality.

Males were prevalent (68%) and the mean age on discovery of LBBB was 37 ± 11 years (median 40 years). In 28%, LBBB was intermittent at the time of discovery, while in the remaining it was stable.

A familial progressive conduction disease proved unlikely in most subjects. Indeed, only 3 (5%) had a familial juvenile LBBB, and a further 2 (4%) had family members aged over 50 years with a familial LBBB. In addition, only 2% had a family history of PM implantation. No patient had a family history of Brugada syndrome, which in some families with SN5A defects may be found in addition to primary conduction disease [18, 19]. However, the absence (or rarity) of a familial progressive cardiac conduction disease in our patients with idiopathic LBBB is not surprising, as Lenegre Disease (when related to SCN5A mutations) more frequently shows first-degree AV block and RBBB (± LAH or LPH) and only seldom LBBB [18, 20].

An obstructive coronary artery disease was excluded in all patients who underwent coronary circulation studies. Indeed, in no patients who had coronary angiography were coronary obstructions detected. In addition, no patient who underwent myocardial scintigraphy during effort displayed ischemic changes.

Interestingly, 2 patients who underwent coronary angiography or CT coronary angiography had an anomalous origin of the left circumflex artery from the right coronary sinus. However, the correlation between an anomalous origin of the left circumflex (CX) artery and LBBB is unclear. Indeed, the left bundle branch has a multivessel blood supply both from the left anterior descending artery (LAD), which perfuses the left anterior fascicle (in addition to the right bundle branch), and from the right coronary artery, which perfuses the left posterior fascicle [13]. We may hypothesize an anomaly of secondary coronary vessels, which are difficult to evaluate by means of angiography, but the most probable explanation is that it was an incidental finding.

Another possible cause of LBBB is localized fibrosis of the basal interventricular septum involving the left bundle. This situation was found in 33% of our patients who underwent cardiac MRI. The cause of such fibrosis is difficult to establish. A familial Lenegre disease seems unlikely (see above), but a non-familial primitive degenerative disease cannot be excluded. Another possible cause is focal myocarditis.

Regarding the prognostic significance of LBBB, the data collected in our cases during follow-up support a favorable outcome in most cases. The quality of life of our patients was not impaired; indeed, the majority of them continued their activity, including sporting activity. No patient suffered SD, and no major events occurred in the vast majority of cases. No patient developed pulmonary or systemic diseases, such as sarcoidosis.

Two patients had syncope or pre-syncope; in these cases, despite negative EPS, a prophylactic PM was implanted. During follow-up, neither of them developed complete AV block. In addition, the PM (programmed in VVI at 40/min) intervened less than 1% of the time. In other words, there was no proof that syncope or pre-syncope was related to a paroxysmal AV block, although this hypothesis cannot be excluded. Certainly, however, these patients did not have a progressive conduction disease.

Some authors [15–17] have suggested that LBBB may cause an induced cardiomyopathy by creating an asynchrony of contraction of the left ventricle. In only one of our patients was this the case. Indeed, only one patient developed a dilated cardiomyopathy, while none of the remaining subjects had any reduction in cardiac function.

## 5. Conclusions

LBBB in the young is a rare and heterogeneous condition. In the majority of cases, no precise pathology can be identified. In a few patients, a familial conduction disease is suggested. In several cases, localized fibrosis in the septal surface of the left ventricle is found, which may be the result of myocarditis or of a primitive non-familial conduction disease. In a minority of cases, an anomalous origin of the CX artery is found, which may be an incidental finding.

In the vast majority of cases, the prognosis is good. However, as only 18% of our patients were 60 years old or more at the end of follow-up, we cannot establish the prognosis in older age-groups.

The final suggestion that emerges from our study is that young subjects with idiopathic LBBB should be thoroughly investigated (by MRI, CT coronary angiography, etc.), as many surprises may arise. In addition, long-term clinical follow-up is reasonable in these patients, as progression of the disease in the elderly is not easily predictable.

### 5.1. Limitations

In no case was a genetic test performed to investigate mutations of SCN5A or LMNA, which may be present in progressive cardiac conduction disease [14]. The main reason was that most patients were enrolled many years ago, when genetic tests were difficult to perform. In any case, as specified above, LBBB was not familial in the vast majority of cases; moreover, PM implantation was very rare in relatives of our patients. Consequently, a familial genetic cardiac conduction disease seems unlikely in the majority of cases.

## Figures and Tables

**Table 1:** Baseline characteristics of patients: yrs = years; IHD = ischemic heart disease; PM = pacemaker; SD = sudden death; DCM = dilated cardiomyopathy; ^*∗*^ patients also with familial IHD or LBBB.

	*n*. (%)
N. cases	56
Males	38 (68%)
Age on discovery of LBBB	37 ± 11 median 40
Family history of	
IHD	8 (14%)
LBBB	5 (8.9%)
PM	1 (1.7%)
SD	3^*∗*^ (5.2%)
DCM	1 (1.7%)
Stable LBBB on discovery of LBBB	40 (72%)
Intermittent LBBB on discovery of LBBB	16 (28%)
AQRS <−30°	37 (66%)
AQRS ≥−30°	19 (34%)
QRS duration (m sec)	149 ± 17

**Table 2:** Echocardiographic parameters on enrollment and during follow-up. Data during follow-up do not include those of the patient who developed a dilated cardiomyopathy. Abbreviations: LVEDVi = indexed left ventricular end-diastolic volume; LVESVi = indexed left ventricular end-systolic volume; LVEF = left ventricular ejection fraction; E cm/s = early mitral inflow velocity; A cm/s = late-diastolic mitral inflow velocity; Longitudinal LVdys = left ventricular dyssynchrony index; IVMD = interventricular mechanical delay; SPMWD = septal to posterior wall motion delay; P non-significant (NS) when >0.05

	Basal echocardiogram *N* = 57	Echocardiogram during follow-up *N* = 56	*P*
LVEDVi (Ml/m^2^)	78 ± 15	80 ± 14	NS
LVESVi (Ml/m^2^)	33 ± 8	34 ± 7	NS
LVEF (%)	59 ± 6	58 ± 9	NS
E (cm/s)	54 ± 16	53 ± 15	NS
A (cm/s)	81 ± 16	80 ± 15	NS
Longitudinal LV (dys)	39 ± 28	30 ± 26	NS
Pre-ejection interval of LV (ms)	225 ± 24	223 ± 27	NS
IVMD (ms)	62 ± 21	61 ± 20	NS
SPMWD (ms)	71 ± 27	70 ± 26	NS

**Table 3:** Follow-up data. ^*∗*^One patient suffered accidental death.

Mean follow-up duration ± SD (yrs)	12 ± 10
Median age at the end of follow-up (yrs)	48
Stable LBBB *n* (%)	53/56 (95%)
Intermittent LBBB *n*. (%)	3/56 (5%)
Events during follow-up *n*. (%)	
Death^*∗*^	0 (0%)
SD	0 (0%
PM implantation	2 (3.5%)
Heart failure	1 (1.7%)
Acute myocardial infarction	1 (1.7%)

## Data Availability

Data-sheets are available at Pietro Delise's travel office.
